# High expression of Snail and NF-κB; predicts poor survival in Chinese hepatocellular carcinoma patients

**DOI:** 10.18632/oncotarget.13891

**Published:** 2016-12-10

**Authors:** Min Zhang, Xin Dong, Dengcai Zhang, Xiaojie Chen, Xinyu Zhu

**Affiliations:** ^1^ Department of Pathology, Department of Scientific Research Gansu Provincial Hospital, Lanzhou 730000, P.R. China; ^2^ Department of Scientific Research, Gansu Provincial Hospital, Lanzhou 730000, P.R. China; ^3^ The Third Hospital of Gansu Province, Lanzhou 730020, P.R. China; ^4^ Department of Pathology, Gansu Provincial Maternity and Child-Care Hospital, Lanzhou 730050, P.R. China

**Keywords:** hepatocellular carcinoma, Snail, NF-κB, survival, Chinese population

## Abstract

In this study, we explored the roles of Snail and NF-κB in hepatocellular carcinoma (HCC). Samples of HCC tumor tissue were collected from 83 Chinese HCC patients. Snail and NF-κB expression was then examined based on immunohistochemical staining, and the relations between Snail and NF-κB expression and the clinical characteristics of the patients were assessed using Cox model analysis. Snail and NF-κB were both expressed in HCC tissue, and their levels were strongly correlated. In addition, levels of both Snail and NF-κB expression were negatively related to tumor differentiation, which was an independent factor predictive of survival in HCC patients. Snail and NF-κB may thus be useful markers of tumor differentiation and survival in HCC, and may also be useful for guiding treatment and exploring molecular mechanisms.

## INTRODUCTION

Characterized by a high rate of recurrence and poor prognosis, hepatocellular carcinoma (HCC) is a severe health problem worldwide [1, 2]. Despite various treatment options, including surgical resection, local ablation, radiotherapy and chemotherapy, HCC is associated with a high mortality rate and remains an intractable illness [3]. It is therefore very important to further explore the molecular mechanisms involved in HCC to identify factors useful for the development of effective therapies that improve patient survival.

The zinc-finger transcriptional repressor Snail reportedly contributes to epithelial-to-mesenchymal transition (EMT) in HCC and plays a key role in tumorigenesis, differentiation, migration and invasiveness [4–7]. For example, Snail reportedly downregulates E-cadherin and upregulates MMP-2 expression, thereby promoting invasiveness of human HCC [8]. In addition, NF-κB is another key mediator promoting proliferation, differentiation and invasiveness in tumors [9–11]. It has been reported that Apigenin, a naturally occurring compound, may inhibit EMT in human HCC by inhibiting the NF-κB/Snail pathway [12]. In an earlier study, we showed that Snail and p65 are overexpressed in HCC tissue [13]. However, their predicted impact on the prognosis of Chinese HCC patients remains unclear.

In this study, therefore, we examined the association between Snail and NF-κB expression and tumor differentiation, which was an independent factor predictive of survival in HCC patients.

## RESULTS

### Patient characteristics

**Figure 1:**
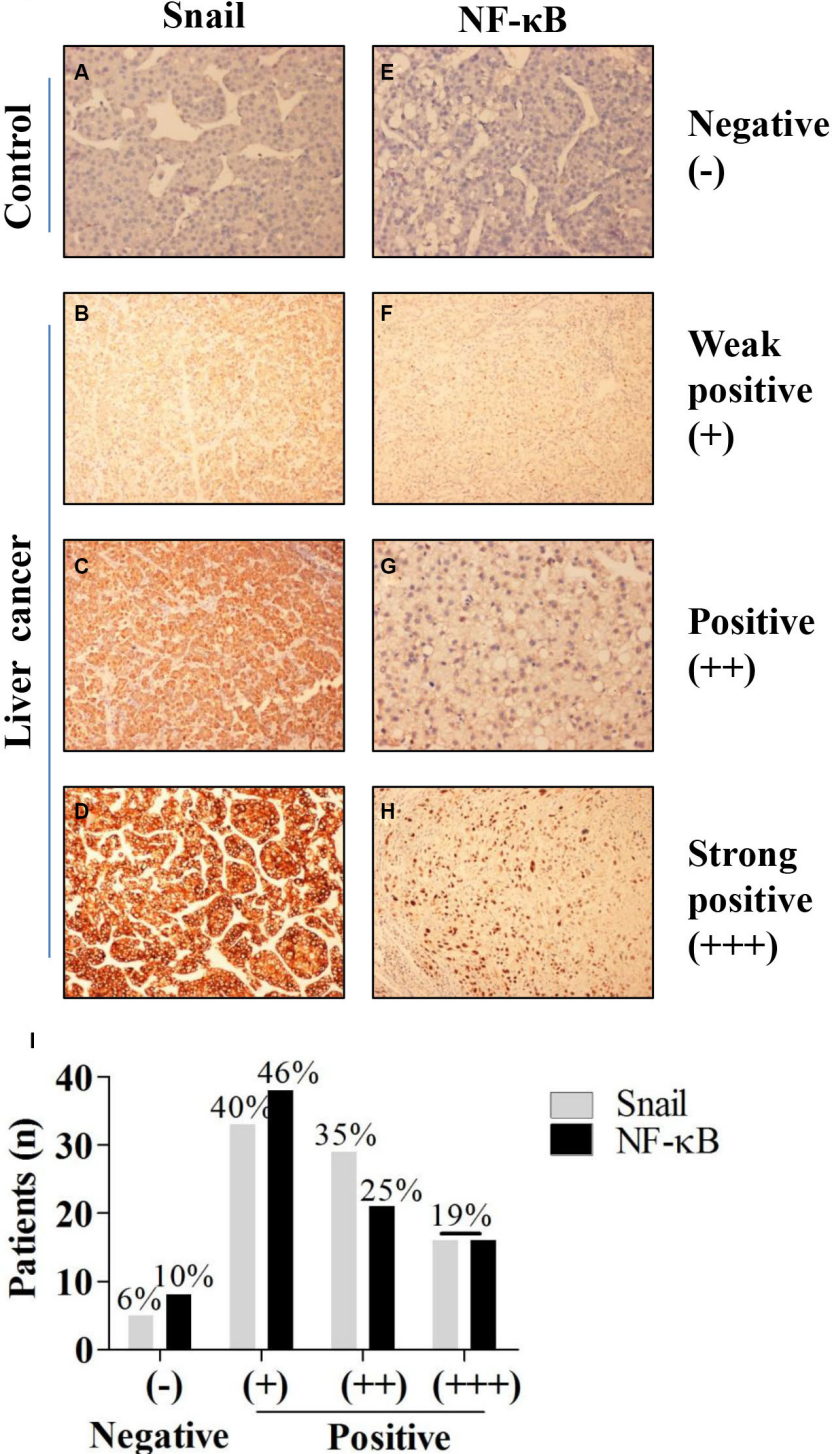
Expression of Snail in HCC tissues (**A**–**D**). Immunohistochemical staining showing expression of Snail in non-tumor liver tissues and in highly differentiated, moderately differentiated, and poorly differentiated HCC tissues. (**E**–**H**). Immunohistochemical staining showing expression of NF-κB in non-tumor liver tissues and in highly differentiated, moderately differentiated and poorly differentiated HCC tissues. (**I**) Rates of Snail and NF-κB staining intensity in HCC patients.

Among the 83 patients (67 male and 16 female) who met the inclusion criteria for this study, the mean age was 51.93 ± 1.488 years. Expression of Snail and NF-κB was increased in patients with HCC The immunohistochemical results summarized in Figure 1 show that normal liver tissue was negative for both Snail and NF-κB staining, whereas HCC tissue stained positively for both factors. Both Snail and NF-κB were localized in the nucleus of hepatocytes. In addition, levels of Snail were closely related to those of NF-κB (Table 1). About 94% of HCC patients were Snail-positive, while 90% were NF-κB-positive (Figure 1I).

**Table 1:** Relation between Snail and NF-κB expression in HCC patients

Snail	NF-κB				Total (*n*)
	0	1	2	3	
0	5	0	0	0	5
1	2	31	0	0	33
2	1	7	21	0	29
3	0	0	0	16	16
Total (*n*)	8	38	21	16	83

*r_s_* = 0.859, *P* < 0.01 (Spearman correlation analysis).

### Snail and NF-κB expression was closely related to tumor differentiation and patient survival

**Figure 2:**
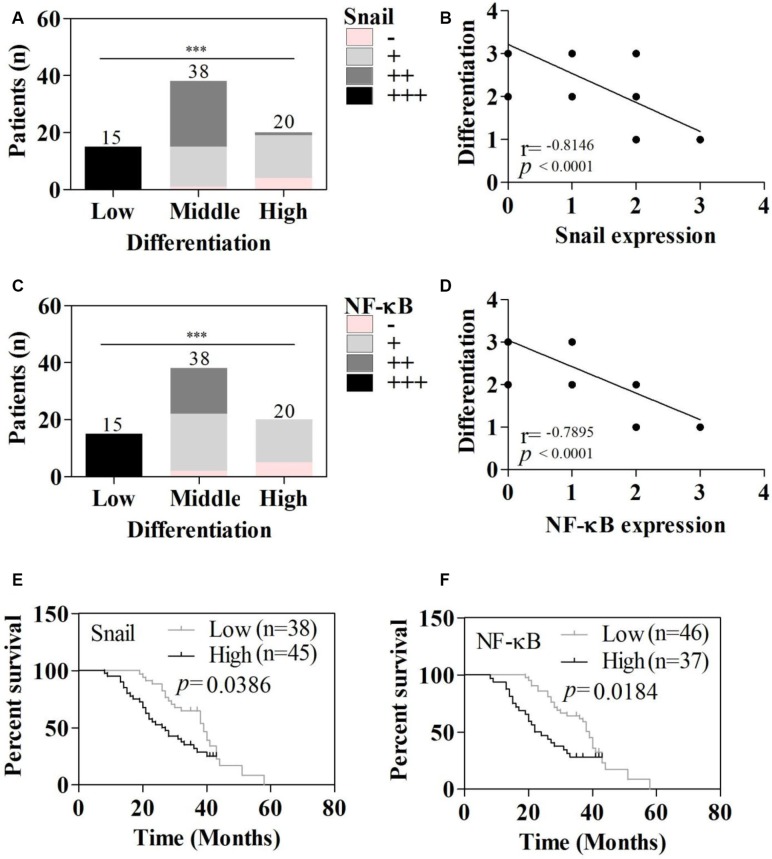
Relations between differentiation, survival, and Snail and NF-κB expression (**A**) Snail staining intensity in HCC tissues with the indicated degree of differentiation. (**B**) Relationship between Snail staining intensity and HCC differentiation. (**C**) NF-κB staining intensity in HCC tissues with the indicated degree of differentiation. (**D**) Relationship between NF-κB staining intensity and HCC differentiation. (**E**) Relationship between Snail staining intensity and survival. (**F**) Relationship between NF-κB staining intensity and survival. *P < 0.05, **P < 0.01, ***P < 0.001.

The intensity of both Snail and NF-κB staining was negatively related to differentiation of HCCs (Figure 2B and 2D). The strongest intensity staining (+++) of Snail and NF-κB was seen in the poorly differentiated group, while weak staining (+) was seen in the highly differentiated group (Figure 2A and 2C).

The results in Figure 2E show that patients with tumors expressing low levels of Snail survived longer than those with tumors expressing high levels of Snail. Similarly, patients with low tumoral NF-κB expression survived longer than with high tumoral NF-κB expression (Figure 2F). Mean survival times were 38.36 months (95% CI, 34.54–42.19) and 29.20 months (95% CI, 25.76–32.64) in the low and high Snail expression groups, respectively, and 37.96 months (95% CI, 34.42–41.50) and 28.42 months (95% CI, 24.42–32.41) in the low and high NF-κB low expression groups (Table 2).

**Table 2:** Survival time (months) of HCC patients expressing high or low levels of Snail or NF-κB

Level	Snail		NF-κB	
	Mean (95% CI)	Median (95% CI)	Mean (95% CI)	Median (95% CI)
Low	38.36 (34.54–42.19)	39.0 (36.74–41.26)	37.96 (34.42–41.50)	39.0 (37.04–40.96)
High	29.20 (25.76–32.64)	28.0 (20.58–35.42)	28.42 (24.42–32.41)	27.0 (16.27–37.73)

### Differentiation is an independent variable closely related to patients’ survival

Univariate Cox analyses indicated that differentiation, tumor size, portal vein tumor thrombus (PVTT), metastasis, and levels of Snail and NF-κB expression were all significant factors associated with patient survival in HCC (Table 3). By contrast, gender, age, tumor number and AJCC stage were not associated with survival of HCCs patients. In addition, multivariate Cox analysis indicated that differentiation is the only independent factor significantly affecting survival in HCC.

**Table 3:** Univariate and multivariate cox analyses of survival in patients with HCC

Tumor features	B	S.E.	Wald-*χ*^2^	*P*	RR (95% CI)
Univariate					
Gender	0.096	0.355	0.073	0.787	1.10 (0.549–2.205)
Age	0.004	0.011	0.153	0.696	1.004 (0.983–1.026)
Differentiation	−0.694	0.201	11.915	0.001	0.500 (0.337–0.741)
Tumor size	0.134	0.043	9.519	0.002	1.143 (1.050–1.244)
Tumor number	0.065	0.157	0.172	0.679	1.067 (0.785–1.452)
PVTT	0.785	0.343	5.230	0.022	2.192 (1.119–4.294)
Metastasis	0.785	0.343	0.523	0.022	2.192 (1.119–4.294)
AJCC	0.248	0.184	1.811	0.178	1.282 (0.893–1.840)
Snail	0.530	0.285	7.843	0.005	1.699 (1.010–2.970)
NF-κB	0.548	0.285	9.453	0.002	1.730 (1.137–3.027)
Multivariate					
Differentiation	−0.694	0.201	11.915	0.001	0.500 (0.337–0.741)

## DISCUSSION

In this study, we assessed Snail and NF-κB expression in tissue samples from patients with HCC and examined the relationship between Snail and NF-κB expression, and between each of those factors and patient survival. We found that both Snail and NF-κB were expressed in HCC tissues and that their expression levels were good predictors of the degree of tumor differentiation and of patient survival. Our observation that nearly all HCC patients are positive for both Snail and NF-κB is consistent with earlier studies [8, 13, 14–16]. Previous *in vitro* studies also showed that activation of NF-κB signaling increases Snail expression [6, 12, 13, 17], which is consistent with our finding that levels of Snail expression correlate closely with those of NF-κB expression in human HCC samples. Taken together, the earlier *in vitro* findings and our present *in vivo* observations strongly suggest that Snail and NF-κB are key mediators in HCC [8, 9, 18].

We found that there is a significant negative relation between Snail and NF-κB and tumor differentiation status, which is an independent factor affecting survival of HCC patients. These results are in agreement with earlier studies showing that by promoting EMT, Snail and NF-κB signaling accelerates proliferation, metastasis and invasiveness in cancer [8, 15, 18–21]. Snail and NF-κB may thus be predictive indicators of prognosis and useful for guiding therapy in HCC patients.

This study also has the following limitations. (1) Sample size is not very large, which means there could be bias. (2) We did not co-localize Snail and NF-κB using immunofluorescent labeling. (3) Additional *in vitro* studies are necessary.

## MATERIALS AND METHODS

### Patient and clinical data

Eighty-three consecutive HCC patients surgically treated at Gansu Provincial Tumor Hospital between June 1, 2008 and January 31, 2013 were the subjects of this study. Included were patients who underwent initial surgery for HCC and whose entire records were available. Patients whose records were not complete were excluded. Patients whose surgical resection was not curative were excluded from the survival analysis. Samples of benign liver tissue adjacent to the tumor tissue were taken from 10 patients as controls.

Clinical data, including gender, age, tumor size, tumor number, metastasis, differentiation, PVTT and JACC stage were collected from the patients’ records in hospital. Survival data were obtained through follow-up. Overall survival was defined as the interval between the date of surgery and death. The maximum follow-up period in this study was 5 years. Follow-up of patients who died of non-liver-related diseases was censored at the time of death.

### Pathology and immunohistochemistry

All pathology reports and tissue slides were reviewed by two pathologists independently. In each section, five visual fields were analyzed, including the upper right, upper left, bottom left, bottom right and middle. Tumor location and size, histologic grade, presence or absence of micro- or macrovascular invasion, and intrahepatic metastasis were re-evaluated. Tumor differentiation was defined as low, moderate, or high according to the Edmondson grading system [22]. Briefly, highly differentiated HCC exhibited cohesive fragments with characteristic vascular/endothelial patterns. Moderately differentiated HCC exhibited prominent pleomorphism with atypical naked nuclei. Poorly differentiated HCC exhibited extreme hypercellularity with loose nests and three-dimensional fragments (often gland-like), pleomorphism, macronucleoli, and focal necrosis [23].

The immunohistochemical staining procedure was performed as follows. Four-μm-thick sections from formalin-fixed, paraffin-embedded blocks were dewaxed and rehydrated, and then treated with 3% hydrogen peroxide for 10 min to block endogenous peroxidase activity. The tissue sections were then treated with boiling citric acid buffer (10 mM sodium citrate and 10 mM citric acid) for 10 min to retrieve the antigen, after which they were incubated with primary anti-Snail and anti-NF-κB antibodies (1:100 dilution) overnight at 4°C. The sections were then rinsed in phosphate-buffered saline (PBS) and incubated with EnVision polymer for 60 min. The sections were stained with freshly prepared diaminobenzidinesolution (DAB) for 8 min, counterstained with hematoxylin, and finally dehydrated and mounted. PBS was substituted for the primary antibodies in the negative controls. To assess the staining of Snail and NF-κB, the cytoplasmic immunoreactive intensity was calculated based on a 0 to 3 scoring system, where 0 = no staining, 1 = weak cytoplasmic staining, 2 = moderate cytoplasmic staining, and 3 = strong granular cytoplasmic staining [24].

### Statistics

Quantitative data are presented with the mean ± standard error and compared using the Student's *t* test. Categorical variables were compared using the *χ*^2^ or Fisher's exact test. Spearman correlation analysis was used to investigate the relationship between Snail and NF-κB immunoreactivity, Snail expression and survival or tumor differentiation, and NF-κB expression and survival or tumor differentiation in HCCs. Overall and disease-free survival curves were generated using the Kaplan-Meier method and compared using the log-rank test. To determine prognostic factors, multivariate regression analysis was performed using the Cox proportional hazards model for variables with *P* < 0.05 in the univariate Cox analyses. Values of *P* < 0.05 were considered significant.

### Ethics

All patients provided written informed consent to use their surgical materials for the study, and the Gansu Provincial Hospital Ethics Committee approved the study. In addition, all procedures in this study were carried out in accordance with the tenets of the Declaration of Helsinki.
